# The Prevalence and Work‐Related Risk Factors of Musculoskeletal Disorders Among Miners at Kalumbila Mine, Kalumbila District Zambia: A Cross‐Sectional Study

**DOI:** 10.1002/hsr2.70907

**Published:** 2025-06-11

**Authors:** Chibuye Kunda, Joseph Lupenga, Chisala D. Meki

**Affiliations:** ^1^ Trident Town Clinic Kalumbia Zambia; ^2^ Department of Environmental Health, School of Public Health University of Zambia Lusaka Zambia; ^3^ Department of Epidemiology & Biostatistics, School of Public Health University of Zambia Lusaka Zambia

**Keywords:** ergonomics, Kalumbila mine, mine workers, mining industry, work‐related musculoskeletal disorders

## Abstract

**Background:**

Mining is a hazardous occupation with high injury rates and work‐related musculoskeletal disorders. However, few studies have reported on the prevalence and risk factors of work‐related musculoskeletal disorders among mineworkers in Zambia.

**Aim:**

The study examined risk factors of work‐related musculoskeletal disorders among mineworkers at Kalumbila mine in Zambia.

**Methods:**

A cross‐sectional study was undertaken, and data were collected using a self‐administered Cornell Musculoskeletal Discomfort Questionnaire. A total of 357 participants were selected using a systematic random sampling method from among the male and female mine workers at the Kalumbila mine. The study used Stata/SE version 17 for univariate and multiple logistic regression analyses. A *p* value of < 0.05 indicated there was a statistically significant result.

**Results:**

A total of 357 mine workers were recruited. The study revealed 275 (77%) of the participants reporting pain or discomfort in at least one body location. The highest level of discomfort or pain was reported in the lower back 184 (51.5%), upper back 90 (25.2%) and, neck 89 (24.9%). In the multivariable analysis, those with tertiary education (AOR 4.27, 95% CI: 1.78–10.24, *p* = 0.001), those who took more than two breaks in a typical working day were 9.51 times more likely to experience WRMSD compared to those who did not take any breaks while working (AOR 9.51, 95% CI: 1.15–79.96, *p* = 0.04). and operating machinery causing whole‐body vibration (AOR 3.17 CI 95%: 1.59–6.34, *p* = 0.001) were associated with work‐related musculoskeletal disorders.

**Conclusion:**

Self‐reported work‐related musculoskeletal disorders were common among mine workers, underscoring the need to invest in worker safety through ergonomic programs and workers' training on safety measures.

## 1 Introduction

Musculoskeletal disorders (MSDs) continue to pose a serious public health burden to the population. The Global Burden of Disease Study 2019 indicates that the global incidence of MSDs in 2019 was 322.75 million (95% uncertainty interval (UI): 292.67– 354.31), with an estimated 117540 (95% UI 94.84–136.05) deaths and 150.08 million (95% UI 108.78–197.82) disability‐adjusted life‐years (DALYs) [1]. In 2019, occupational risks were the leading causes of DALYs of MSDs worldwide, accounting for 15.31 million DALYs (95% UI 10.54–20.76). The impact of these occupational risk factors was particularly pronounced in medium Sociodemographic Index (SDI) regions (4.85 million, 95% UI 3.33–6.61) and low to medium SDI regions (3.32 million, 95% UI 2.29–4.51) [1]. Workers in high‐risk industries, such as mining, where tasks involve repetitive movements, heavy manual Labor, awkward postures, vibration exposure, and long hours, are especially vulnerable to MSDs [2, 3].

When the MSD is associated with occupational risk, the term “work‐related MSDs (WRMSDs)” is frequently used [4]. Work‐related musculoskeletal disorders are a serious issue across many industries, including large‐scale mining operations, as a result WRMSDs are a significant burden among miners [5, 6, 7, 8]. The existing work conditions in mines have the potential to lead to the development of WRMSDs [9]. Studies indicate that miners are exposed to a range of moderate to severe risks associated with WRMSDs [10]. For instance, long‐term exposure to occupational whole‐body vibration has been associated with an array of adverse effects on the body [11]. A systematic review and meta‐analysis of 50 studies found that the prevalence of WRMSDs among miners was highest in the upper back (50.39%, CI 95%: 31.23%–54.73%) and lowest in the knees (16.03%, CI 95%: 11.78%–20.28%) [5].

Although a variety of risk factors have been identified as contributing to WRMSDs, they are a significant challenge to control, particularly in unstructured mine environments [6, 8]. A systematic review and meta‐analysis of 51 studies showed that 23 studies found whole‐body vibration exposure as a causative factor for WRMSDs. Other factors included awkward postures, repetitive movement, demographics, BMI, gender, smoking, and psychological factors [7]. However, specific factors involved may vary based on the type of mine in question and the personal characteristics of the miners involved [6].

The mining industry in Zambia has been a principal contributor to economic expansion over the course of the country's history, and the country's job opportunities [12]. However, the growing number of individuals employed in the mining sector may also result in an elevated prevalence of WRMSDs among miners. While work‐related WRMSDs are prevalent among miners, they haven't received significant academic attention. Few studies comprehensively examine the nature and extent of WRMSDs among miners in Zambia [13, 14], and neighbouring countries such as Zimbabwe [15, 16], the Democratic Republic of Congo [17, 18], Botswana [19], and Namibia [20]. Some of these studies focused solely on low back pain, reporting prevalence rates of 65.52%–78.8% [14, 15, 16], while neglecting other affected body parts. Overall WRMSD prevalence varies across studies in Zambia and neighbouring countries, with estimates ranging from 25% to 42.6% [13, 17, 19, 20, 21]. However, the currently reported rates may not accurately reflect the present situation, as relatively little research has explored the burden of WRMSDs and associated factors in this region. Therefore, this study examined the risk factors that are associated with WMSDs among mineworkers at Kalumbila mine in Zambia to improve the understanding of the burden of WRMSDs among miners.

## 2 Methods

### 2.1 Study Design and Setting

A cross‐sectional study was conducted among mineworkers at the Kalumbila mine in the northwestern province of Zambia. Kalumbila Mine is owned by First Quantum, a global copper company that produces copper in the form of concentrate, cathode and anode, and has reserves of nickel, gold, and cobalt. The Kalumbila open pit copper mine is located 150 km west of Solwezi in the Northwestern Province of Zambia. Data was collected between the November 15, 2022 and completed on January 19, 2023.

### 2.2 Study Population and Sample Size

The study was conducted among mineworkers at Kalumbila Mine, which has 3362 employees spread across departments including Human Resources, Mining Operations, Mining Technical, Health and Safety, Engineering, Process Plant, Commercial, and Site Services. The study included mineworkers aged 18 and above with a minimum of 1 year of work experience and gave informed consent to participate in the study. The sample size was determined using the Yamane sample size formula by Yamane (1967) [22]. The formula is given by:

$$n=\frac{N}{1+N(e^{2})}$$

where: *n* is the sample size, *N* is the population size (3362), *e* is the margin of error (expressed as a decimal = 0.05). A 0.05 margin of error is deemed acceptable due to the actual population proportion of WRMSD residing within the range of 10%–90%, thus yielding a sample size that is sufficient [23, 24].

$$n=\frac{3362}{1+3362{\left(0.05\right)}^{2}}n=357$$

A total of 357 mineworkers were recruited using a stratified random sampling method across five departments: Engineering, Mining Operations, Process Plant, Commercial, Finance and Human Resource. The sample was drawn proportionally, ensuring adequate representation and a more accurate assessment of musculoskeletal disorders among mineworkers.

▪ Mining: 50% of total population, 179 participants.
▪ Engineering: 20% of total population, 71 participants.
▪ Process Plant: 20% of total population, 71 participants.
▪ Commercial Finance and Human Resource: 10% of total population, 36 participants.

### 2.3 Study Variables

The outcome variable was work‐related musculoskeletal disorders defined as the symptoms in the back, neck, shoulder, and knee and wrist in the last 7 days. The explanatory variables considered were categorized into three risk factors associated with WRMSDs as: (i) socio‐demographic factors (age, sex, educational status, marital status and BMI), (ii) physical work factors (awkward posture: working overhead, back bent forward, bending, wrist bent back or forward and twisting, whole‐body vibration and lifting of heavy objects), and (iii) psychosocial work factors (e.g., job control, job satisfaction and job demand).

### 2.4 Data Collection

The data collection tool consisted of four sections: first section assessed socio‐demographic factors, second section assessed physical work environment, third section assessed psychological factors and the last section assessed WRMSDs among participants. The questions on the three sections (socio‐demographic, physical work, environment and psychological factors) were based on previous published research studies of WRMSDs among miners. Data on WRMSDs were collected using the Cornell Musculoskeletal Discomfort Questionnaire (CMDQ), developed by Hedge, Morimoto and McCrobie to measure self‐reported musculoskeletal discomfort for all body segments [25]. The questionnaire was developed for research screening purposes and the results can be analysed in four ways: (1) by simply counting the number of symptoms per person; (2) by summing the rating values for each person; (3) by weighting the rating scores to more easily identify the most serious problems as follows: never = 0, 1–2 times/week = 1.5, 3–4 times/week = 3.5, every day = 5, several times every day = 10; and (4) by multiplying the above Frequency score (0, 1.5, 3.5, 5, 10) by the Discomfort score (1,2,3) by the Interference score (1,2,3). As for this study, the questionnaire was analyzed simply by counting the number of symptoms per person. The questionnaire demonstrated a Cronbach's alpha coefficient of 0.986 [26]. The Cronbach's alpha coefficient for all three sections of the frequency of discomfort, severity of discomfort, and interference scales was determined to be 0.955, 0.961 and 0.969, respectively [26]. The questionnaire was designed in English.

### 2.5 Data Analysis

All statistical analyses were conducted using Stata version 17.0 (StataCorp LP, College Station, TX) [27]. The data was entered into excel and imported to Stata software. Frequencies and percentages were used to provide summary description of socio‐demographic, physical work environment and psychological factors. The frequency and proportion of WRMSDs among mine workers and for affected anatomical sites are presented. The distributions of MSD among demographic and work‐related factors were tested using the *χ* ^2^ test. If the expected frequencies in the contingency table were less than 5, a Fisher's exact test was employed. The univariate and multivariable logistic regression analysis was performed to assess the association between potential risk factors (socio‐demographic and physical work‐related factors) and WRMSDs reporting for odds ratios (ORs) and 95% confidence interval (95% CI). The analyses aimed to determine which factors are significantly associated with the development of WRMSDs. Variables with a *p* < 0.30 in the univariate logistic regression analysis were included in the multivariable analysis [28]. To create the final model, the investigator led backward elimination logistic regression was used retaining all explanatory variables with a *p* value < 0.20. The cut‐off level for variable selection was set high so that important variables that can influence the WRMSDs are not missed, and so that less important variables with practical and clinical implications are not deleted [28]. In model selection, the Bayesian information criterion (BIC) and Akaike's information criterion (AIC) were used, with the model with the lowest value being preferred. The Hosmer–Lemeshow test was used to evaluate the goodness of fit of the final model (*p* = 0.77). The presence of multicollinearity was examined by utilizing the Variance Inflation Factor (VIF) (mean = 1.50). A 5% significance level was chosen for all tests, which were carried out as two‐tailed tests. All statistical tests, except for the exploratory comparison of study body region MSD prevalence with global prevalence, were pre‐specified analyses.

### 2.6 Ethical Approval

All procedures were performed under the Declaration of Helsinki, relevant legislation, and institutional guidelines, and were approved by the Research Ethics Committee (REF. No. 2723‐2022). Informed consent was obtained from all participants, and confidentiality was maintained by not including any personal information or identifiers on the questionnaires.

## 3 Results

### 3.1 Demographic and Work‐Related Characteristics

Three hundred and fifty‐seven mine workers participated in the study. Approximately 44% (159/356) of the participants were aged between 30 and 40 years, while 105 (29.5%) of the participants were aged between 41 and 50 years. Males made up the majority of the participants in the study, 295 (83.8%). Musculoskeletal pain discomforts were highest among participants that had tertiary education (85.5%, *p*= 0.002), had three or more breaks at work (93.8%, *p*= 0.04), that spent less than an hour per day standing (84.1%, *p*= 0.001), that spent less than an hour working overhead (80.7%, *p*= 0.03), and those whose job required high‐level skills (80.2%, *p*= 0.037) (Table 1).

**Table 1:** Demographic and work‐related characteristics of the study participants from Kalumbila Mine in Solwezi stratified by musculoskeletal disorder status.

		No discomfort	Discomfort		
Variable	Category	*N* (%)	*N* (%)	Total	*p* value
Age group	20–30	6 (13.3)	39 (86.7)	45	0.13^a^
	30–40	38 (23.9)	121 (76.1)	159	
	40–50	21 (20.0)	84 (80.0)	105	
	50–60	10 (30.3)	23 (69.7)	33	
	60+	6 (42.9)	8 (57.1)	14	
Sex	Female	12 (21.1)	45 (78.9)	57	0.83^b^
	Male	66 (22.4)	229 (77.6)	295	
Marital status	Single	17 (18.9)	73 (81.1)	90	0.31^b^
	Married	64 (24.1)	202 (75.9)	266	
Education	No formal education	3 (50.0)	3 (50.0)	6	**0.002** a
	Primary school	0 (0.0)	1 (100.0)	1	
	Secondary school	24 (36.9)	41 (63.1)	65	
	Tertiary school	21 (14.5)	124 (85.5)	145	
	Trade school	33 (23.7)	106 (76.3)	139	
BMI category	Underweight	0 (0.0)	1 (100.0)	1	0.55^a^
	Healthy	31 (23.0)	104 (77.0)	135	
	Overweight	36 (24.2)	113 (75.8)	149	
	Obese	10 (15.9)	53 (84.1)	63	
Smoking	No	68 (23.1)	227 (76.9)	295	0.70^b^
	Yes	12 (20.7)	46 (79.3)	58	
Work Schedule	Day and night work	46 (20.4)	179 (79.6)	225	0.41^a^
	Days only	33 (26.0)	94 (74.0)	127	
	Night only	0 (0.0)	1 (100.0)	1	
Working hours per day	≤ 8 h for 5 days	3 (15.8)	16 (84.2)	19	0.78^a^
	> 8 h for 5 days	73 (22.2)	256 (77.8)	329	
Break time	None	44 (29.0)	108 (71.0)	152	**0.04** a
	Once	29 (19.5)	120 (80.5)	149	
	Twice	5 (13.9)	31 (86.1)	36	
	≥ 3 times	1 (6.2)	15 (93.8)	16	
Handle vibrating objects	No	60 (21.2)	223 (78.80)	283	0.19^b^
	Yes	20 (28.6)	50 (71.43)	70	
Hand vibrating objects	Chipping machine	8 (44.4)	10 (55.6)	18	0.29^a^
	Hand drills	4 (20.0)	16 (80.0)	20	
	Hand‐held grinders	2 (15.4)	11 (84.6)	13	
	Riveting guns	3 (30.0)	7 (70.0)	10	
Operate vibrating machine	No	56 (24.1)	176 (75.9)	232	0.26^b^
	Yes	23 (18.9)	99 (81.1)	122	
Body vibrating machine	Dozers	5 (21.7)	18 (78.3)	23	0.40^a^
	Excavator	2 (12.5)	14 (87.5)	16	
	Dump truck	6 (12.2)	43 (87.8)	49	
	Others	8 (25.8)	23 (74.2)	31	
Lifting weights	1–5 times	7 (18.92)	30 (81.08)	37	0.52^b^
	> 5 times	3 (37.50)	5 (62.50)	8	
	None	68 (22.22)	238 (77.78)	306	
Hours spent standing	Less than 1 h	28 (16.0)	147 (84.0)	175	**0.001** a
	1–4 h	26 (23.6)	84 (76.4)	110	
	5–8 h	21 (34.4)	40 (65.6)	61	
	More than 9 h	4 (80.0)	1 (20.0)	5	
Hours working overhead	Less than an hour	53 (19.4)	221 (80.7)	274	**0.03** a
	1–4 h	15 (25.0)	45 (75.0)	60	
	5–8 h	6 (50.0)	6 (50.0)	12	
Repetitive work	No	12 (27.9)	31 (72.1)	43	0.23^b^
	Yes	60 (19.9)	242 (80.1)	302	
Fast work	No	12 (28.6)	30 (71.4)	42	0.19^b^
	Yes	59 (19.8)	239 (80.2)	298	
High‐level skills	No	8 (42.1)	11 (57.9)	19	**0.04** a
	Yes	64 (19.8)	260 (80.2)	324	

*Note:* Bold *p* value significant at *p *< 0.05.

^a^ Fisher's Exact test.

^b^ *χ* ^2^ test.

### 3.2 The Prevalence and Common Types of Work‐Related Musculoskeletal Disorders

**Figure 1:**
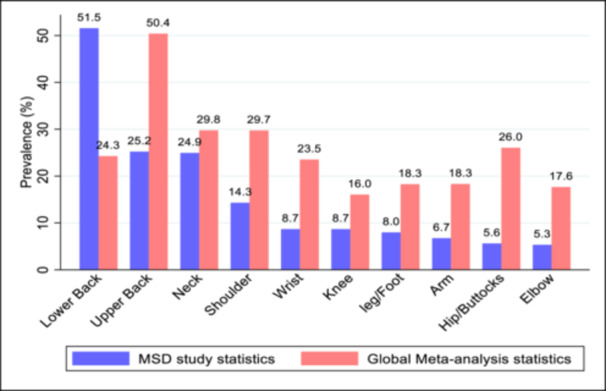
Comparison of musculoskeletal pain prevalence between this study and global meta‐analysis.

In the 7 days preceding questionnaire completion, 77% (95% CI: 72.4–81.1) of the participants reported pain or discomfort in at least one body location. Figure 1 compares the prevalence of musculoskeletal disorders (MSD) pain in this study with a global meta‐analysis [5], revealing variations in WRMSD distribution across body regions. While lower back pain was most prevalent in this study (51.5%), the global meta‐analysis showed the highest prevalence in upper back pain (50.4%). The elbow was the least affected body part in this study (5.3%) compared to the knee in the global statistics (16%). Except for lower back pain, the prevalence rates in this study were lower than those reported globally.

#### 3.2.1 Frequency of the Pain/Discomfort

Participants who experienced pain or discomfort were asked to report the frequency of the pain or discomfort. Most participants reported experiencing pain 1–2 times per week in their right foot (50%), left lower leg (57.9%), left knee (52.9%), left wrist (57.1%), right wrist (58.6%), left forearm (72.7%), right forearm (66.7%), lower back (58.7%), left upper limb (62.5%), right upper limb (60.0%), upper back (71.1%), left shoulder (55.6%), right shoulder (54.5%) and neck (79.8%). Table 2 shows the information on the frequency of the pain/discomfort experienced by the study participants.

**Table 2:** Frequency of pain, severity of the discomfort, and interference with work among study participants from Kalumbila Mine in Solwezi.

	Frequency of pain/discomfort					Severity of discomfort				Interference of discomfort with work			
Anatomical site	1–2 times last week	3–4 times last week	Once everyday	Several times every day	Total	Moderately uncomfortable	Slightly uncomfortable	Very uncomfortable	Total	Not at all	Slightly interfered	Substantially interfered	Total
Neck	71 (79.8)	8 (9.0)	3 (3.4)	7 (7.9)	89	33 (37.1)	50 (36.2)	6 (6.7)	89	50 (53.8)	35 (37.6)	8 (8.6)	93
Shoulder (right)	24 (6.7)	13 (3.6)	4 (1.1)	3 (0.8)	44	26 (50.0)	19 (36.5)	7 (13.5)	52	24 (42.9)	19 (33.9)	13 (23.2)	56
Shoulder (left)	15 (4.2)	7 (2.0)	2 (0.6)	3 (0.8)	27	15 (39.5)	20 (52.6)	3 (7.9)	38	24 (57.1)	13 (31.0)	5 (11.9)	42
Upper back	64 (17.9)	10 (2.8)	12 (3.4)	4 (1.1)	90	56 (62.9)	26 (29.2)	7 (7.9)	89	20 (22.5)	58 (65.2)	11 (12.4)	89
Upper arm (right)	12 (3.4)	2 (0.6)	3 (0.8)	3 (0.8)	20	9 (31.0)	18 (62.1)	2 (6.9)	29	21 (60.0)	13 (37.1)	1 (2.9)	35
Upper arm (left)	10 (2.8)	2 (0.6)	2 (0.6)	2 (0.6)	16	7 (28.0)	16 (64.0)	2 (8.0)	25	21 (65.6)	9 (28.1)	2 (6.3)	32
Lower back	108 (30.3)	40 (11.2)	13 (3.6)	23 (6.4)	184	103 (56.3)	42 (23.0)	38 (20.8)	183	31 (17.4)	93 (52.2)	54 (30.3)	178
Forearm (right)	10 (2.8)	3 (0.8)	0 (0.0)	2 (0.6)	15	7 (28.0)	18 (72.0)	0 (0.0)	25	19 (73.1)	6 (23.1)	1 (3.8)	26
Forearm (left)	8 (2.2)	2 (0.6)	1 (0.3)	0 (0.0)	11	3 (17.6)	14 (82.4)	0 (0.0)	17	20 (90.9)	1 (4.5)	1 (4.5)	22
Wrist (right)	17 (4.8)	5 (1.4)	2 (0.6)	5 (1.4)	29	12 (37.5)	16 (50.0)	4 (12.5)	32	15 (41.7)	16 (44.4)	5 (13.9)	36
Wrist (left)	8 (2.2)	3 (0.8)	2 (0.6)	1 (0.3)	14	6 (27.3)	15 (68.2)	1 (4.5)	22	18 (69.2)	3 (11.5)	5 (19.2)	26
Hip/buttocks	7 (2.0)	7 (2.0)	2 (0.6)	4 (1.1)	20	10 (35.7)	13 (46.4)	5 (17.9)	28	17 (56.7)	8 (26.7)	5 (16.7)	30
Thigh (right)	3 (0.8)	4 (1.1)	3 (0.8)	4 (1.1)	14	9 (37.5)	13 (54.2)	2 (8.3)	24	17(65.4)	8 (30.8)	1 (3.8)	26
Thigh (left)	4 (1.1)	2 (0.6)	2 (0.6)	2 (0.6)	10	7 (33.3)	13 (61.9)	1 (4.8)	21	18 (78.3)	5 (21.7)	0 (0.0)	23
Knee (right)	14 (3.9)	9 (2.5)	1 (0.3)	5 (1.4)	29	12 (31.6)	14 (36.8)	12 (31.6)	38	17 (41.5)	9 (22.0)	15 (36.6)	41
Knee (left)	9 (2.5)	4 (1.1)	3 (0.8)	1 (0.3)	17	6 (22.2)	14 (51.9)	7 (25.9)	27	17 (56.7)	3 (10.0)	10 (33.3)	30
Lower leg (right)	8 (2.2)	7 (2.0)	2 (0.6)	2 (0.6)	19	4 (14.3)	23 (82.1)	1 (3.6)	28	23 (74.2)	7 (22.6)	1 (3.2)	31
Lower leg (left)	11 (3.1)	6 (1.7)	1 (0.3)	1 (0.3)	19	5 (18.5)	21 (77.8)	1 (3.7)	27	21 (70.0)	7 (23.3)	2 (6.7)	30
Foot (right)	10 (2.8)	7 (2.0)	2 (0.6)	1 (0.3)	20	5 (17.9)	17 (60.7)	6 (21.4)	28	20 (66.7)	7 (23.3)	3 (10.0)	30
Foot (left)	6 (1.7)	5 (1.4)	1 (0.3)	1 (0.3)	13	3 (14.3)	16 76.2)	2 (9.5)	21	19 (76.0)	4 (16.0)	2 (8.0)	25

#### 3.2.2 Severity of the Discomfort

The severity of discomfort was also reported by participants who experienced pain or discomfort, as shown in Table 2. Most of the participants experienced moderate discomfort in the right shoulder 24 (60%), left shoulder 12 (50.0%), the upper back 54 (65.9%), right upper arm 8 (50%), lower back 100 (57.5%), Hip 8 (50.0%), right 7 (58.3%) and left 5 (55.6%) thighs, and right 12 (52.2%) and left 6 (54.5%) wrists. Mild discomfort was most commonly reported in the neck 44 (54.3%), left upper arms 6 (50.0%), right 6 (54.6%) and left 3 (50.0%) forearms, right 12 (75.0%) and left 12 (75.0%) lower leg, and left foot 7 (77.8%).

#### 3.2.3 Interference of the Pain/Discomfort With Work

The participants were asked about the extent to which the pain or discomfort they experienced interfered with their work, as shown in Table 2. Most participants with upper back 54 (68.4%), lower back 91 (52.9%), left thigh 5 (62.5%), right thigh 7 (63.6%), right wrist 14 (56.0%), right forearm 6 (54.6%) and right upper arm 11 (61.1%) pain/discomfort reported that the pain/discomfort interfered slightly with their work. On the other hand, participants who had pain on the left knee, 9 (56.3%), indicated that it substantially interfered with their work.

### 3.3 Factors Associated With Musculoskeletal Disorders Among Mine Workers

To identify factors associated with WRMSDs among mine workers, both univariate and multiple logistic regression analyses were performed. A number of socio‐demographic factors, work‐related factors, and psychological factors were included in the regression model. In the univariate analysis, age, educational level, working long hours while standing, hours spent working overhead, and having a job that requires high skills were associated with WRMSD among mine workers (*p *< 0.05). In the multivariable analysis, those with tertiary education (AOR 4.27, 95% CI: 1.78–10.24, *p*= 0.001), and those with trade education (AOR 2.16, 95% CI: 1.02–4.61, *p*= 0.05) were more likely to have WRMSD than those with secondary education. Those who took more than two breaks in a typical working day were 9.51 times more likely to experience WRMSD compared to those who did not take any breaks while working (AOR 9.51, 95% CI: 1.15–79.96, *p*= 0.04). Participants who operated machinery causing whole‐body vibration were 3.17 times more likely to experience WRMSD than those who did not (AOR 3.17 CI: 95%: 1.59–6.34, *p*= 0.001) (Table 3).

**Table 3:** Factors associated with WMSDs among mineworkers at Kalumbila mine, Kalumbila District.

Variable	Crude (CI 95%)	*p* value	Adjusted (CI 95%)	*p* value
Age				
20–30	ref			
30–40	0.49 (0.19–1.25)	0.13		
40–50	0.62 (0.23–1.65)	0.33		
50–60	0.35 (0.11–1.10)	0.07		
60 and above	0.21 (0.05–0.80)	0.02		
Sex				
Female	ref			
Male	0.93 (0.46–1.85)	0.83		
Marital status				
Divorced/single/widowed	ref			
Married	0.73 (0.40–1.34)	0.31		
Educational level				
Secondary education	Ref			
Tertiary school	3.46 (1.74–6.85)	< 0.001	4.27 (1.78– 10.24)	0.001
Trade school	1.88 (0.99–3.56)	0.05	2.16 (1.02–4.61)	0.05
Smoking				
No	ref			
Yes	1.15 (0.56–2.29)	0.70		
Body mass index				
Healthy	ref			
Overweight	0.94 (0.54–1.62)	0.81		
Obese	1.58 (0.72–3.47)	0.25		
Length of service in years	0.94 (0.85–1.03)	0.17		
Working schedule				
Days only	Ref			
Days and night work	1.37 (0.82–2.28)	0.23		
Hours worked per day				
< 8 h for 5 days	Ref			
> 8 h for 5 days	0.66 (0.19–2.32)	0.51		
Breaks on a typical working day				
None	Ref			
Once	1.68 (0.99–2.88)	0.06	1.74 (0.93–3.25)	0.09
Twice	2.53 (0.92–6.92)	0.07	2.85 (0.78–10.42)	0.11
More than three times	6.11 (0.78– 47.68)	0.08	9.51 (1.13– 79.96)	0.04
Handle objects that make the hand vibrate				
No	Ref			
Yes	0.67 (0.37–1.22)	0.19		
Operate machinery that causes whole‐body vibration				
No	Ref			
Yes	1.37 (0.79–2.36)	0.26	3.17 (1.59–6.34)	0.001
Lifting objects weighing > 25 kg on a typical workday				
None	Ref			
1–5 times	1.22 (0.52–2.91)	0.65		
> 5 times	0.48 (0.11–2.04)	0.32		
Hours spent working standing				
Less than an hour	Ref			
1–4 h	0.62 (0.33–1.12)	0.11		
5–8 h	0.36 (0.19–0.71)	0.003		
More than 9 h	0.05 (0.01–0.44)	0.007		
Hours spent working overhead				
Less than an hour	Ref			
1–4 h	0.72 (0.37–1.39)	0.33		
5–8 h	0.24 (0.07–0.77)	0.02		
The job involves a lot of repetitive work				
No	Ref			
Yes	1.56 (0.76–3.22)	0.23		
The job requires a high level of skill				
No	Ref			
Yes	2.95 (1.14–7.64)	0.03		
The job requires working very fast				
No	Ref			
Yes	1.62 (0.78–3.35)	0.19	2.09 (0.94–4.63)	0.07

*Note:* Model statistics, Hosmer–Lemeshow test, *p* value = 0.77; Variance inflation factor (VIF) mean value = 1.50; Pseudo *R* ^2^ = 0.0875.

## 4 Discussion

The aim of the study was to assess the prevalence and work‐related risk factors of WRMSDs among mine workers. The study found that 77% of the participants had pain in at least one part of their body. The highest levels of discomfort or pain were reported in the lower back, upper back, and neck. Associated with WRMSDs in miners were tertiary and trade education, taking more than two breaks in a typical working day, and operating machinery that produces whole‐body vibration.

The WRMSD was found to be very common among mine workers, with 77% of participants reporting pain or discomfort in at least one body site over a 7‐day period before the study. The findings highlight the significant occupational health challenges faced by individuals employed in the mining sector. This underscores the urgent need for comprehensive occupational health interventions and ergonomic improvements in the mining industry to address and mitigate the high prevalence of WRMSDs among mine workers. The study's findings confirm previous observations from multiple studies that suggest mine workers are associated with an increased prevalence of WRMSD [29, 30, 31]. The reported prevalence in this study is much higher when compared with that reported in previous studies conducted in China among construction workers (57.9%) [32], in Pakistan among construction workers (52%) [33], in Democratic republic of Congo among gold mine workers (25%) [17], and in India among surface miners (44.2%) [34]. On the other hand, a much higher prevalence than that reported in this study is reported in Ghana among workers in the gold mining industry (85.5%) [31]. Although these observational studies show a high prevalence of WRMSDs, the prevalence varies between studies, depending on the population, location, and data collection methods.

The most common area of pain was the lower back, followed by the upper back and neck. These findings support findings from previous studies suggesting that lower back pain is the most common problem among miners [17, 31, 33, 34]. Lower back pain is associated with the number of hours worked in a shift and the repetitive movements of a body part [35]. This study reports a prevalence of 51.5%, which is consistent with the findings of Yong et al. [30], who reported a prevalence of 50.7%, but is higher than that reported in the study from the Democratic Republic of Congo (14.8%) [17]. The high prevalence of lower back pain among miners indicates that ergonomic programmes need to be strengthened to improve and protect their health.

In contrast to the findings of this study on the most affected part of the body, a study of mining truck drivers found that the most common musculoskeletal complaints were in the neck, lower back, knees, and right shoulder [29]. Another study also found that the weekly prevalence of MSDs in coal mine workers was associated with the waist, followed by the neck and shoulder [30]. Additionally, another study found that the highest prevalence of WRMSD symptoms was in the neck, followed by the shoulder, upper back, and lower back [32].

It was revealed in the study that most people reported moderate discomfort in the right shoulder, upper back, and lower back, while mild discomfort was reported in most parts of the body. The severity of discomfort reported in different body regions suggests a pattern of strain that may be directly linked to specific mining tasks and work postures. These findings could inform targeted ergonomic interventions, such as redesigning tools or modifying work processes to reduce strain on the most affected areas. In contrast to this study, one study found that the highest rates of moderate pain were found in the elbows, lower back, and ankles/feet [10]. Although most participants reported experiencing pain in most parts of the body 1–2 times a week, a high number of people reported that these complaints either slightly or significantly interfered with their work, most commonly upper and lower back pain/discomfort. Similar to these study findings, studies from China and Ghana also reported that back pain was associated with more absenteeism from work among miners [30, 31].

The study also reported on the work‐related risk factors of WRMSDs among mine workers. Mine workers with a tertiary education and trade education were found to be at a higher risk of developing WRMSD than those with a secondary education. This finding is consistent with previous findings that show a relationship between higher education and an increased likelihood of developing WRMSD [17, 36]. Although the underlying reasons for the observed results are unknown, it is reasonable to hypothesize that mine workers with postsecondary education, whether tertiary or trade‐based, may undertake a disproportionate number of tasks demanding repetitive movements or awkward postures, leading to an increased risk of developing WRMSDs. To fully understand this relationship and develop effective prevention strategies for at‐risk miners, further investigation into the underlying causes is necessary.

The study found that workers who took more than two breaks in a typical working day were more likely to experience WRMSD compared to those who did not take any breaks while working. This finding is in contrast to what exists in the literature, as the use of breaks during working hours is recommended as an intervention that could reduce the risk of WRMSD among workers [37]. An inadequate number of rest breaks during the working day was found to be a significant predictor of musculoskeletal disorders [38, 39]. On the contrary, findings from low‐quality evidence indicate a potential lack of association between break type and participant‐reported musculoskeletal pain, discomfort, and fatigue [40]. Because this study's cross‐sectional design only captured a single moment in time, it's possible that participants who took more breaks already had MSDs, leading to a higher frequency of breaks. This unexpected result might also be explained by several potential biases. Because of pain and discomfort, workers with WRMSD may have reported their break‐taking habits differently than those without symptoms, leading to self‐reporting errors. Recall bias and measurement errors in defining what constitutes a break could further distort the data. As such, this finding needs to be interpreted with caution and suggests future research should use objective measures and longitudinal designs to clarify the relationship between break‐taking and WRMDs.

The study revealed that mine workers who operated machinery causing whole‐body vibration were at an increased risk of developing WRMSD compared to those who did not operate such machinery. This finding is supported by the findings of the study, which showed that dump truck operators, dozer operators, and grader operators were most at risk of developing WRMSD [34]. These findings further contribute to the evidence suggesting that vibration exposure among operators and drivers is significantly associated with musculoskeletal disorders, including lower back pain [7, 41, 42]. To reduce vibration‐related risks, ergonomic interventions like vibration‐dampening seats, proper vehicle suspension systems [6], and work schedules can be implemented. Worker training on optimal seating posture and machine handling can also help [43]. Conducting regular maintenance checks and a combination of engineering controls and administrative measures can further mitigate risks [44].

Socio‐demographic factors such as age, sex, length of service, body mass index, and smoking were not statistically associated with WRMSD in this study. This is in contrast to the findings of previous studies, which found that age [29, 32, 45], sex [45], body mass index [46], length of service [30, 32, 45], and smoking were associated with WRMSDs in workers [45]. Work involving repetitive movements was not associated with WRMSD in this study, which is contrary to previous studies that found repetitive movements to be associated with WRMSD [30]. Working shifts was not associated with WRMSDs, which conflicts with the findings of a previous study, which found that workers working two shifts had an increased risk of experiencing WRMSDs than those working a fixed day shift [45]. Considering that only a few factors were associated with WRMSDs in this study, there is still a need for further studies to provide a better understanding.

Although the study provides important information about WRMSD, it has some methodological limitations. The study used self‐report measures, which are prone to social desirability effects, so it is possible that the reported prevalence is overestimated. Second, the study design is not an ideal design for determining causal effects since outcome and exposure were measured simultaneously. For example, it's possible that participants with two or more breaks have already suffered from MSDs, rather than being at higher risk. The prevalence of WRMSD may be exaggerated because some problems may have been caused by other nonwork‐related things not recorded in the study, such as falls and injuries that occurred in nonwork settings.

### 4.1 Conclusion

The prevalence of WRMSDs is relatively high among mine workers with more than three‐quarters experiencing pain/discomfort in at least one part of the body. Lower back disorders were the most common WRMSDs among miners, followed by upper back and neck disorders, and these disorders were also found to cause the most work disability. These findings underscore the importance of investing and strengthening worker safety through ergonomic programs such as regular health assessments, workload rotation, adjustable workstations, scheduled micro‐breaks, and workers' training on safety measures to reduce the burden of work‐related musculoskeletal disorders among miners. In addition, great attention should be paid to the factors associated with WMSD among miners, including those with postsecondary education and those who operate vibrating machinery. There is need for longitudinal studies to track WRMSD progression over time.

## Author Contributions

**Chibuye Kunda:** writing – original draft, investigation, conceptualization, writing – review and editing, methodology, validation, data curation. **Joseph Lupenga:** software, formal analysis, data curation, validation, writing – review and editing, writing – original draft. **Chisala D. Meki:** supervision, writing – review and editing.

## Ethics Statement

All procedures were performed under the Declaration of Helsinki, relevant legislation and institutional guidelines, and were approved by the University of Zambia Biomedical Research Ethics Committee (REF. No. 2723‐2022).

## Consent

Informed consent was obtained from all participants, and confidentiality was maintained by not including any personal information or identifiers on the questionnaires.

## Conflicts of Interest

The authors declare no conflicts of interest.

## Transparency Statement

The lead author, Chibuye Kunda, affirms that this manuscript is an honest, accurate, and transparent account of the study being reported; that no important aspects of the study have been omitted; and that any discrepancies from the study as planned (and, if relevant, registered) have been explained.

## Data Availability

The data that support the findings of this study are available from the corresponding author upon reasonable request.
